# Effect of Acupuncture on Functional Capacity and Health-Related Quality of Life of Hemodialysis Patients: Study Protocol for a Randomized Controlled Trial

**DOI:** 10.3390/healthcare10102050

**Published:** 2022-10-17

**Authors:** Marta Correia de Carvalho, Jorge Pereira Machado, Manuel Laranjeira, José Nunes de Azevedo, Pedro Azevedo

**Affiliations:** 1 ICBAS—Instituto de Ciências Biomédicas Abel Salazar, Universidade do Porto, 4050-313 Porto, Portugal; 2 CBSin—Center of BioSciences in Integrative Health, 4000-105 Porto, Portugal; 3 INC—Instituto de Neurociências, 4100-141 Porto, Portugal; 4 TECSAM—Tecnologia e Serviços Médicos SA, 5370-530 Mirandela, Portugal

**Keywords:** chronic kidney disease, hemodialysis, acupuncture, functional capacity, health-related quality of life, integrative medicine, randomized controlled trial

## Abstract

The proposed randomized controlled trial protocol will evaluate the effect of acupuncture treatment on the functional capacity (FC) and health-related quality of life (HRQOL) in chronic kidney disease (CKD) with glomerular filtration rate (GFR) category 5 (CKG G5) patients receiving maintenance dialysis. Patients undergoing hemodialysis (HD) from a dialysis center will be randomly assigned to experimental, placebo and control groups. In order to determine the difference between the same number of treatments performed three times or one treatment a week, experimental (verum acupuncture) and placebo (sham acupuncture) groups will receive a total of nine acupuncture treatments; however, both groups will be divided into subgroups A and B. The same selection of acupuncture points will be applied to both experimental subgroups and the placebo subgroups will receive acupuncture on non-acupuncture points. The results will be assessed by the 6-min Walk Test, Handgrip Test, 30-sec Sit-to-Stand and Kidney Disease Quality of Life-Short Form and will be held at baseline, after treatment and 12 weeks post-treatment follow up. This paper describes the rationale and design for a randomized, patient-assessor blinded controlled trial, which may provide evidence for the clinical application of acupuncture in CKG G5 patients undergoing HD.

## 1. Introduction

Over the last few decades, chronic diseases have become increasingly relevant and, according to the statistics, this type of disease is the leading cause of death worldwide and considered by the World Health Organization (WHO) to be the greatest threat to the health of the population in the 21^st^ century [1].

Chronic kidney disease (CKD) is considered a public health problem due to the increasing incidence and prevalence, as well as to high associated economic costs, substantially affecting the life of the patient and implying a significant burden on the health system [2,3]. Kidney Disease: Improving Global Outcomes (KDIGO) 2012 Clinical Practice Guideline for the Evaluation and Management of Chronic Kidney Disease defines CKD as abnormalities of kidney structure or function, present for more than 3 months, with implications for health and classified based on cause, glomerular filtration rate (GFR < 60 mL/min/1.73 m^2^ and albuminuria (AER ≥ 30 mg/24 h; ACR ≥ 30 mg/g (≥3 mg/mmol)) categories. Glomerular filtration rate (GFR) is generally accepted as the best overall index of kidney function and decreased GFR is associated with a higher risk of complications of CKD [4,5].

Although CKD etiology is diverse and due to diseases, such as glomerulopathies, polycystic kidney disease, autoimmune diseases, systemic infections, recurrent urinary tract infections, obstructive uropathies and neoplasms, CKD is most commonly associated with diabetes and hypertension, which are determining factors in the increase and prevalence of the disease [6]. Complications of decreased kidney function can lead to kidney failure (GFR < 15 mL/min/1.73 m^2^), the most advanced stage of CKD. Patients who have CKD with GFR category 5 (CKG G5) can only be treated by kidney replacement therapy (KRT), in particular peritoneal dialysis (PD), hemodialysis (HD), kidney transplantation (KT) and conservative kidney management [4].

The improvement in technologies and innovation around hemodialysis (HD) treatment has contributed to the increase in kidney failure prevalence due to the fact that patients survive longer on dialysis [7,8]. Patients undergoing HD suffer from several physical and emotional problems, have a complex and demanding treatment regimen, face a stressful and disruptive chronic illness with high impact on their quality of life [4,9,10] and have a significant decrease in functional capacity (FC) [11].

Traditional Chinese Medicine (TCM), recognized by the WHO and increasingly accepted in Western medicine as a therapeutic approach to the treatment of various diseases, is based on different concepts of etiopathogenesis, semiology, physiology and therapeutics. Scientific advances, especially in the field of neurosciences, have decisively contributed to the clarification and understanding of the neurobiological basis inherent in the acupuncture mechanisms of action [12,13].

Recent studies report early research for the application of TCM concepts and acupuncture in the field of nephrology. Examples include a study conducted by Paterno et al. (2008) suggested that there can be beneficial effects of electroacupuncture and moxibustion on CKD [14]; Kim et al. (2011) studied patients undergoing hemodialysis and receiving acupuncture treatment for their symptoms, and acupuncture seemed to be feasible and safe for symptom management [15]; Bullen et al. (2018) concluded that acupuncture during HD may contribute toward improvements in health-related quality of life [16]; Yu et al. (2017) evaluated the feasibility effect of acupuncture on renal function in patients with CKD and results suggested reduced creatinine levels and increased GFR [17].

Despite encouraging results, more scientific research will be required to expand understanding of the mechanisms responsible for positive action and of the therapeutic effects of acupuncture [18]. According to Kim et al. (2016), future high-quality randomized controlled trials are required to verify the safety and effectiveness in pre-dialysis patients with CKD and in those undergoing dialysis, to compare acupuncture with placebos and to verify whether intensive short-term acupuncture interventions or ongoing but less frequent treatments are advantageous for patients [19].

The fundamental questions guiding our entire research project are: What is the effect of TCM therapeutic strategies in the improvement of symptoms resulting from renal replacement therapy in CKG G5 patients undergoing HD? Does acupuncture have a positive effect on FC and health-related quality of life (HRQOL) in hemodialysis patients? If it does, are the results maintained over the long term? In addition, what is the effectiveness of short-term intensive interventions when compared to less frequent and prolonged interventions? Is it possible to integrate acupuncture into the dialysis care routine? Given our research project and the complexity of CKD, the objectives of this study are: (1) to assess the effect of acupuncture on FC and HRQOL in patients undergoing hemodialysis; (2) to evaluate the specific effects of acupuncture as compared to a placebo; (3) to evaluate short- and long-term effects of acupuncture; (4) to determine the difference between short-term intensive and ongoing but less frequent acupuncture treatments; (5) to assess the feasibility of integrating acupuncture in dialysis care.

## 2. Materials and Methods

### 2.1. Study Setting

**Figure 1:**
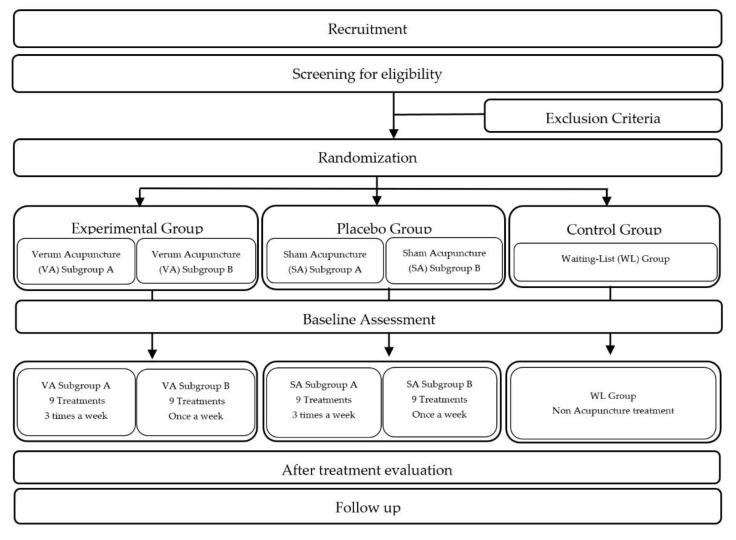
Trial Flow Chart.

A parallel-group patient-assessor blinded randomized controlled trial will be conducted in a single center and according to the trial flow chart procedure presented in Figure 1. The recruitment and the whole treatment process will be carried out at the Hemodialysis Center of TECSAM—Tecnologia e Serviços Médicos, SA, in Mirandela, Portugal. The research protocol and informed consent procedure were approved by the Ethics Committee of Centro Hospitalar Universitário do Porto/Instituto Ciências Biomédicas Abel Salazar (registered number 2019/CE/P026_P304/2019/CETI), and is an integral part of the research project of the Doctoral Program in Biomedical Sciences of ICBAS School of Medicine and Biomedical Sciences, Oporto University, Portugal. This trial was registered at ClinicalTrials.gov (NCT05362643). The study protocol will be reported in accordance with the Standard Protocol Items: Recommendations for Interventional Trials (SPIRIT) guidelines [20].

### 2.2. Recruitment

An advertisement will be posted on the clinic notice board and if all study criteria are met, volunteer patients who are receiving treatment at TECSAM Hemodialysis Center will be invited to participate.

### 2.3. Eligibility Criteria

Inclusion and exclusion criteria will be applied during the participant selection process and potentially eligible participants will be identified by a nephrologist and through screening of their TECSAM Hemodialysis Center patient medical records.

This trial will include male and female patients, age 18 years or older, who have been receiving regular hemodialysis treatment for more than 3 months, undergoing sessions 3times a week, at 4 h per session, and who are in a medically stable condition.

Patients who refuse to participate in the study or who have a clinical indication that prevents their participation in the study, and patients with other comorbidities, such as poorly controlled malignant hypertension, unstable angina, uncontrolled diabetes mellitus, cerebrovascular failure with recurrent syncope, uncontrolled heart failure, severe mental illness or cognitive impairment, will be excluded. Those with other relevant circumstances, including the inability to practice physical exercise, having undergone acupuncture treatment in the past two weeks, having experienced a known hypersensitivity reaction and/or other side effects after acupuncture treatment, and displaying an inability to cooperate with the procedures inherent to the application of the procedure, will be also excluded from the study.

### 2.4. Randomization and Allocation

After screening for eligibility, a written informed consent form containing an explanation of the objectives and procedures for the research project’s implementation will be obtained from each individual who agrees to participate, in compliance with the revised version of the Declaration of Helsinki and the Oviedo Convention. The procedure will be performed by an independent assessor, with each one assigned an anonymous code. Participants will be randomly allocated in a 1:1:1 ratio and assigned to the experimental group (verum acupuncture), the placebo group (sham acupuncture) or the control group (non-acupuncture) following simple randomization procedures employing a computerized random number list. The randomization sequence will be created using Microsoft^®^ Excel^®^ for Microsoft 365 MSO Microsoft, Washington, USA by an independent researcher who will prepare the assignments in opaque envelopes and take charge of concealing the allocation sequence.

After the random assignment of the participants into the three groups and regarding the frequency of treatment, the first 12 randomized patients in the verum acupuncture (VA) and sham acupuncture (SA) groups will receive treatment 3 times a week, and the remaining 12 will receive treatment once a week, with a total of 9 treatments for each subgroup.

### 2.5. Blinding

The participants, the outcome assessor, the statistician and the Traditional Chinese Medicine practitioner will be blind to the allocation. Regarding the intervention itself, due to its specific nature, only the Traditional Chinese Medicine practitioner will not be blind to the verum acupuncture, sham and non-acupuncture groups.

To assess the effectiveness of the blinding, after the treatment, a questionnaire will be administered to ask the participants in the VA and SA groups what sort of acupuncture treatment (verum or sham) they believe they received. This question will allow the calculation of the James Blinding Index (BI) [21,22].

### 2.6. Intervention

The experimental (verum acupuncture) and placebo (sham acupuncture) groups will receive 9 acupuncture treatments. Both groups will be divided into two subgroups with a different treatment frequency: subgroup A will receive 3 acupuncture sessions per week over 3 weeks; and subgroup B will receive 1 acupuncture session per week over 9 weeks. The same selection of acupuncture points, identified according to the method of point location issued by the WHO [23], will be applied to both experimental subgroups A and B. Placebo subgroups A and B will receive acupuncture at the same non-specific acupuncture points. Details of the acupuncture intervention will be described according to the revised Standards for Reporting Interventions in Clinical Trials of Acupuncture (STRICTA) 2010 Checklist [24], as listed in Table 1.

## 3. Outcome Measurement

### 3.1. Primary Outcome

The primary outcome measure will be the change in the parameters of FC and HRQOL. FC will be measured by a 6-Minute Walk Test (6MWT) [28]. The 6MWT assesses the submaximal level of functional exercise capacity and is simple and convenient to carry out. Changes in the distance walked in 6 minutes will be used to evaluate the efficacy of therapeutic interventions.

Quality of Life will be assessed by Kidney Disease Quality of Life-Short Form (KDQOL-SFTM 1.3) [29]. The KDQOL-SF contains eight generic subscales that assess various aspects of HRQOL and has been widely used in CKD.

### 3.2. Secondary Outcomes

Secondary outcome measures will be changes in handgrip and lower-limb strength. Handgrip strength will be measured by a Hand Grip Strength (HGS) test [30,31], using a digital dynamometer. The HGS test is an indicator of overall muscle strength and is commonly used.

**Figure 2:**
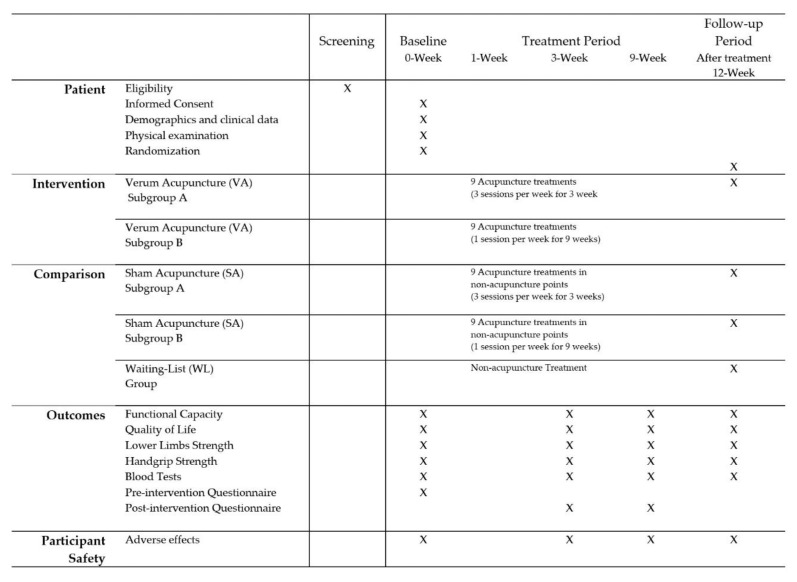
Timing of visits and data collection.

Lower-limb strength will be measured by a 30 s Sit-to-Stand (30STS) test [32]. Demographic, clinical variables and blood analytical data will be collected using a Sociodemographic and Clinical Data Collection Form in the baseline period. Pre- and post-intervention questionnaires will be applied before and after the treatment period. The outcome measurement time points are detailed in Figure 2.

### 3.3. Participant Timeline

The timeline for the study visits, enrolment process, interventions, assessments, and follow-ups carried out on the participants will be reported based on the Standard Protocol Items: Recommendations for Interventional Trials (SPIRIT) guidelines [20], as shown in Figure 2.

### 3.4. Statistical Analysis and Sample size

Demographic and clinical data for each group at baseline will be compared using Fishers’ Exact Test (categorical variables) and one-way ANOVA (continuous variables). Two-way repeated-measures ANOVA with interaction between time (baseline, 3 weeks, 9 weeks and follow-up period 12 weeks after treatment) and group will be used to assess the effect of the treatment. Differences over time within each group and subgroup will be assessed with repeated-measures ANOVA, and differences among groups at each moment will be evaluated with one-way ANOVA. A significance level of 5% will be used; differences will be considered statistically significant for *p*-value < 0.05. Statistical analysis will be performed with IBM SPSS Statistics software, version 27.0, IBM, New York, NY, USA [33].

For the sample size, a minimum of 20 patients in each group (experimental, placebo and control groups) was estimated to achieve a small to medium effect size (f = 0.19) [34] in the two-way repeated-measures ANOVA, with a statistical power of 80% and a significance level of 5%. Sample-size calculations were carried out using G*Power V.3.1 [35]. Assuming a possible dropout rate of 15%, a minimum of 24 patients will be randomly selected for each group. In the experimental and placebo groups, half of the patients (*n* = 12) will be placed in subgroup A, with three acupuncture sessions per week over 3 weeks, and half (*n* = 12) will be placed in subgroup B, with one acupuncture session per week over 9 weeks.

### 3.5. Adverse Events Assessment

Regarding patient safety, any adverse event related to the acupuncture treatment will be observed and reported by patients, practitioners or care providers during each patient visit. Although the participants in the study will not need medical assistance in the acupuncture treatments, which will occur during hemodialysis sessions, they will be under permanent medical surveillance. If any adverse reaction occurs during the intervention, emergency measures will be taken as appropriate.

### 3.6. Quality Control and Data Collection

Before the clinical trial begins, the research team will receive specialized training to ensure the quality of the study is maintained. Topics concerning the research process, eligibility criteria, data collection and management and assessment of adverse events will be covered.

Screening for eligibility, demographic and clinical-data collection and physical examinations will be performed by the TECSAM clinical team, acting as an independent assessor. The main researcher, the practitioner who will provide the acupuncture treatments, will not have access to patient data until anonymization, randomization and allocation procedures are complete. Baseline, after-treatment and follow-up outcome variables will be assessed by the TECSAM head nurse, who will not know to which group each subject is assigned. Moreover, the statistician will be unaware of the allocation details during the research period, to ensure that the objectivity and impartiality of the data are guaranteed.

The clinical trial will be stopped in case of serious adverse events. Participants will be withdrawn from the study if they are reluctant to continue or if they ask to be excluded.

## 4. Discussion

The protocol described above refers to a parallel-group patient-assessor blinded randomized clinical trial planned to assess the effect of acupuncture compared with placebo-sham acupuncture and non-acupuncture groups. Therefore, it was designed following the Standards for Reporting Interventions in Clinical Trials of Acupuncture (STRICTA) [24] and reported with the Standard Protocol Items: Recommendations for Interventional Trials (SPIRIT) guidelines [20].

CKD is a debilitating disease, and standards of medical care involve aggressive monitoring for signs of disease progression and early referral to specialists for dialysis or possible renal transplant [36]. As kidney disease progresses, patients often experience a variety of symptoms. The initiation of dialysis can have a variable effect on quality-of-life measures and on the alleviation of uremic signs and symptoms, such as anorexia, fatigue, cognitive impairment, depressive symptoms, pruritus and sleep disturbance [37].

HRQOL has been widely accepted as a valid marker of treatment outcomes and mortality for patients with chronic diseases [38]. Hemodialysis negatively impacts the HRQOL of patients, affecting their physical, functional, metabolic, social and mental conditions, with a high impact on functional capacity when compared to healthy individuals [39,40,41].

Many pharmacological interventions for people with CKD have known risks of adverse events and, according to the literature, acupuncture is commonly used in symptom management for chronic diseases as well as in palliative care [42]. However, the safety and efficacy of acupuncture for people with CKD remains largely unknown. Considering the aims of our study, we expect that verum acupuncture compared to sham acupuncture and non-acupuncture will improve the functional capacity of hemodialysis patients, as evidenced by an increase in the distance walked on the 6MWT and an increase in scores for quality of life. In addition, we expect an increase in handgrip strength and an improvement in peripheral muscle strength. According to Kim et al. (2016), there was low-quality evidence of the short-term effects of manual acupressure as an adjuvant intervention for fatigue, depression, sleep disturbance and uremic pruritus in patients undergoing regular hemodialysis [19]. Under this assumption, this study protocol was designed to check the effect of acupuncture after treatment and at 12 weeks post-treatment. In this way, we expect that the effect of acupuncture will be maintained in the follow-up period.

Some studies, especially in the pain field, suggest that three treatments per week obtain better results than one treatment per week [43,44]. We also expect to see better results with more intensive interventions compared to less frequent and prolonged interventions.

Following review of the literature and to the best of the authors’ knowledge, there are currently no comparable studies. Therefore, our trial seems to be an innovative study for exploring the effect of acupuncture and the frequency of treatment on improving FC and HRQOL in patients undergoing HD. Furthermore, supported by only a few clinical cases reported (personal data) prior to the design and development of the research project, it is possible to predict efficacy and the advantages of integrating acupuncture treatments into routine hemodialysis as a complementary therapy to conventional treatments.

Despite fulfilling strict methodological criteria, it is possible to identify limitations in our study protocol. First, the sample size; second, the TCM practitioner performing the acupuncture treatment will not be blinded due to the nature of the intervention; third, comorbidities inherent to chronic kidney disease may lead to a high dropout rate during the follow-up period.

## 5. Conclusions

In conclusion, this study protocol provides a standardized procedure for conducting our randomized clinical trial. Despite its limitations, we expect the results to provide evidence for the efficacy of acupuncture on FC and HRQOL, as well as for further research to validate the therapeutic effects of acupuncture in patients undergoing HD.

## Figures and Tables

**Table 1:** Details of acupuncture treatment.

Item	Detail	
1. Acupuncture Rationale	(1a) Style of acupuncture	Manual acupuncture.
	(1b) Reasoning for treatment provided, based on historical context, literature sources, and/or consensus methods, with references where appropriate.	The protocol treatment provided is based on traditional meridian theory, literature sources, clinical experience and consensus method of experts in Traditional Chinese Medicine, Acupuncture and Nephrology [25,26,27], members of research team.
	(1c) Extent to which treatment was varied	Experimental (verum acupuncture) group will receive a total of 9 acupuncture treatments, however, will be divided into subgroups A and B. Experimental subgroup A will receive three acupuncture sessions per week over 3 weeks and subgroup B will receive one a week for 9 weeks. The same choice of acupuncture points will be applied to both experimental subgroups A and B.
2. Details of Needling	(2a) Number of needle insertions per subject per session	A total of 5 fixed acupoints and a total of 8 needle insertions per subject and per session.
	(2b) Names of points used	*Taixi* (KI3), bilateral; *Sanyinjiao* (SP6), bilateral; *Zusanli* (ST36), bilateral; *Shenmen* (HT7) unilateral, in the arm without arteriovenous fistula; *Guanyuan* (CV4), unilateral. Acupuncture points will be found according to the WHO points location method.
	(2c) Depth of insertion, based on a specified unit of measurement, or on a particular tissue level	After local area disinfection with alcohol wipes, acupoints CV4, ST36, KI3 and SP6 will be inserted perpendicularly (15 to 20 mm depth) and HT7 inserted slightly obliquely (10 mm depth).
	(2d) Response sought	*De qi* sensation (described as a compositional sensation including numbness, soreness, distention, heaviness) will be achieved through lifting, thrusting and twirling manipulations.
	(2e) Needle stimulation	Manual stimulation. After generating a needling sensation, needles will be manipulated for one minute every ten minutes during needle retention.
	(2f) Needle retention time	25 min.
	(2g) Needle type	Sterilized stainless-steel needle (0.25 × 25 mm) Tewa, asia-med GmbH, Kirchplatz 1, 82049 Pullach, Germany.
3. Treatment Regimen	(3a) Number of treatment sessions	A total of 9 treatment sessions for both groups.
	(3b) Frequency and duration of treatment sessions	Verum Acupuncture Subgroup A: three times a week for 3 weeks. Verum Acupuncture Subgroup B: once a week for 9 weeks.
4. Other Components of Treatment	(4a) Details of other interventions administered to the acupuncture group	Apart from the usual care routine for hemodialysis sessions, during the study period, no further intervention will be allowed.
	(4b) Setting and context of treatment, including instructions to practitioners, and information and explanations to patients	Treatment sessions will take place at the Hemodialysis Center of TECSAM Tecnologia e Serviços Médicos, SA, in Mirandela, during hemodialysis sessions. Participants will be informed about the entire procedure and a clarification meeting will also be held with the hemodialysis clinical team before treatments starts.
5. Practitioner Background	(5) Description of participating acupuncturists	M.C.d.C is a licensed specialist in Traditional Chinese Medicine with professional card number C-006513 issued by the Regulator of Health System Central Administration in Portugal, ACSS (Administração Central do Sistema de Saúde, I.P.); five years of professional experience; a Traditional Chinese Medicine Master’s degree from Instituto de Ciências Biomédicas Abel Salazar, Universidade do Porto, Portugal; completion of Advanced Chinese Medicine Course from Guangzhou University of Chinese Medicine, Guangzhou, P.R. China.
6. Control or Comparator Interventions	(6a) Rationale for the control or comparator in the context of the research question, with sources that justify this choice	Participants will randomly be assigned to experimental group (verum acupuncture), placebo group (sham acupuncture) or control group (waiting list).
	(6b) Precise description of the control or comparator. If sham acupuncture or any other type of acupuncture-like control is used, provide details as for Items 1 to 3 above.	The placebo group (sham acupuncture) will receive a total of 9 acupuncture treatments, however, will be divided into subgroups A and B. Placebo subgroup A will receive three acupuncture sessions per week over 3 weeks and subgroup B will receive one a week for 9 weeks. Manual acupuncture will be performed as superficial needling (5 mm depth) at non-acupuncture points without an attempt to achieve a *De qi* sensation and without stimulation, lasting 25 min, using a sterilized stainless-steel needle (0.25 × 25 mm; Tewa, asia-med GmbH, Kirchplatz 1, 82049 Pullach, Germany).The control group (waiting list) will not receive any acupuncture treatment during the study period. **List of Non-Acupuncture points used and their location** In the present study, the non-acupuncture points selected will be the points located on the non-meridian but near the acupuncture points described above. Non-Acupuncture point 1 (NA1) Located near KI3 (*Taixi*). On the posteromedial aspect of the ankle, at the midpoint of KI3 (*Taixi*) and (*Fuliu*). KI7 (*Fuliu*) is located on the posteromedial aspect of the leg, anterior to the calcaneal tendon, 2 *cun* superior to the prominence of the medial malleolus. Non-Acupuncture point 2 (NA2) Located near SP6 (*Sanyinjiao*). On the leg, at the midpoint of the medial side of the tibia, 1 *cun* distal from SP6 (*Sanyinjiao*). Non-Acupuncture point 3 (NA3) Located near ST36 (*Zusanli*). On the anterior aspect of the leg, at midpoint of ST37 (*Shangjuxu*) and the Gallbladder Meridian. ST37 (*Shangjuxu*) is located on the anterior aspect of the leg, on the line connecting ST35 with ST41, 6 *cun* inferior to ST35, on the tibialis anterior muscle. Non-Acupuncture point 4 (NA4) Located near HT7 (*Shenmen*). On the wrist, at the transverse crease of the wrist, at the midpoint of HT7 (*Shenmen*) and PC7 (Daling), between the Pericardium Meridian and the Heart Meridian. Non-Acupuncture point 5 (NA5) Located near CV4 (*Guanyuan*). On the lower abdomen, 1 *cun* lateral from CV6 (*Qihai*). CV6 (*Qihai*) is located 1.5 *cun* inferior to the center of the umbilicus, on the anterior median line.

## Data Availability

Not applicable.

## References

[1] WHO (2014). Global Status Report on Noncommunicable Diseases 2014.

[2] Foundation N.K. (2002). K/DOQI clinical practice guidelines for chronic kidney disease: Evaluation, classification, and stratification. Am. J. Kidney Dis..

[3] Wang V., Vilme H., Maciejewski M.L., Boulware L.E. (2016). The economic burden of chronic kidney disease and end-stage renal disease. Semin. Nephrol..

[4] Kidney Disease: Improving Global Outcomes (KDIGO) CKD Work Group (2013). KDIGO 2012 clinical practice guideline for the evaluation and management of chronic kidney disease. Kidney Int. Suppl..

[5] de Boer I.H., Caramori M.L., Chan J.C., Heerspink H.J., Hurst C., Khunti K., Liew A., Michos E.D., Navaneethan S.D., Olowu W.A. (2020). KDIGO 2020 clinical practice guideline for diabetes management in chronic kidney disease. Kidney Int..

[6] Jha V., Garcia-Garcia G., Iseki K., Li Z., Naicker S., Plattner B., Saran R., Wang A.Y., Yang C.W. (2013). Chronic kidney disease: Global dimension and perspectives. Lancet.

[7] Liew A. (2018). Perspectives in renal replacement therapy: Haemodialysis. Nephrology.

[8] Golshayan D., Pascual M. (2019). Burden of end-stage renal disease and evolving challenges in kidney transplantation. Transpl. Int..

[9] Kittiskulnam P., Sheshadri A., Johansen K.L. (2016). Consequences of CKD on functioning. Semin. Nephrol..

[10] Goh Z.S., Griva K. (2018). Anxiety and depression in patients with end-stage renal disease: Impact and management challenges—A narrative review. Int. J. Nephrol. Renovasc. Dis..

[11] Painter P. (2005). Physical functioning in end-stage renal disease patients: Update 2005. Hemodial. Int..

[12] Napadow V., Ahn A., Longhurst J., Lao L., Stener-Victorin E., Harris R., Langevin H.M. (2008). The status and future of acupuncture mechanism research. J. Altern. Complement. Med..

[13] Ifrim Chen F., Antochi A.D., Barbilian A.G. (2019). Acupuncture and the retrospect of its modern research. Rom. J. Morphol. Embryol..

[14] Paterno J.C., Freire A.O., Soares M.F., Franco M.F., Schor N., Teixeira V.P. (2008). Electroacupuncture and moxibustion attenuate the progression of renal disease in 5/6 nephrectomized rats. Kidney Blood Press. Res..

[15] Kim K.H., Kim T.H., Kang J.W., Sul J.U., Lee M.S., Kim J.I., Shin M.S., Jung S.Y., Kim A.R., Kang K.W. (2011). Acupuncture for symptom management in hemodialysis patients: A prospective, observational pilot study. J. Altern. Complement. Med..

[16] Bullen A., Awdishu L., Lester W., Moore T., Trzebinska D. (2018). Effect of acupuncture or massage on health-related quality of life of hemodialysis patients. J. Altern. Complement. Med..

[17] Yu J.S., Ho C.H., Wang H.Y., Chen Y.H., Hsieh C.L. (2017). Acupuncture on renal function in patients with chronic kidney disease: A single-blinded, randomized, preliminary controlled study. J. Altern. Complement. Med..

[18] Garcia G.E., Ma S.X., Feng L. (2005). Acupuncture and kidney disease. Adv. Chronic. Kidney Dis..

[19] Kim K.H., Lee M.S., Kim T.H., Kang J.W., Choi T.Y., Lee J.D. (2016). Acupuncture and related interventions for symptoms of chronic kidney disease. Cochrane Database Syst. Rev..

[20] Chan A.W., Tetzlaff J.M., Gøtzsche P.C., Altman D.G., Mann H., Berlin J.A., Dickersin K., Hróbjartsson A., Schulz K.F., Parulekar W.R. (2013). SPIRIT 2013 explanation and elaboration: Guidance for protocols of clinical trials. BMJ.

[21] James K.E., Bloch D.A., Lee K.K., Kraemer H.C., Fuller R.K. (1996). An index for assessing blindness in a multi-centre clinical trial: Disulfiram for alcohol cessation–A VA cooperative study. Stat. Med..

[22] Kolahi J., Bang H., Park J. (2009). Towards a proposal for assessment of blinding success in clinical trials: Up-to-date review. Community Dent. Oral Epidemiol..

[23] Lim S. (2010). WHO standard acupuncture point locations. Evid. Based Complement. Altern. Med..

[24] MacPherson H., Altman D.G., Hammerschlag R., Youping L., Taixiang W., White A., Moher D. (2010). Revised standards for reporting interventions in clinical trials of acupuncture (STRICTA): Extending the CONSORT statement. PLoS Med..

[25] Maciocia G. (2015). The Foundations of Chinese Medicine. A Comprehensive Text.

[26] Li W., Frierman D. (2003). Clinical Nephrology in Chinese Medicine.

[27] Matos L.C., Machado J.P., Monteiro F.J., Greten H.J. (2021). Understanding traditional chinese medicine therapeutics: An overview of the basics and clinical applications. Healthcare.

[28] ATS Committee on Proficiency Standards for Clinical Pulmonary Function Laboratories (2002). American Thoracic Society. ATS statement: Guidelines for the six-minute walk test. Am. J. Respir. Crit. Care Med..

[29] Hays R.D., Kallich J., Mapes D., Coons S., Amin N., Carter W.B., Kamberg C. (1997). Kidney Disease Quality of Life Short Form (KDQOL-SF &trade).

[30] Bobos P., Nazari G., Lu Z., MacDermid J.C. (2020). Measurement properties of the hand grip strength assessment: A systematic review with meta-analysis. Arch. Phys. Med. Rehabil..

[31] Bohannon R.W. (2017). Test-retest reliability of measurements of hand-grip strength obtained by dynamometry from older adults: A systematic review of research in the PubMed database. J. Frailty Aging.

[32] Figueiredo P.H.S., Veloso L.R.S., Lima M.M.O., Vieira C.F.D., Alves F.L., Lacerda A.C.R., Lima V.P., Rodrigues V.G.B., Maciel E.H.B., Costa H.S. (2021). The reliability and validity of the 30-seconds sit-to-stand test and its capacity for assessment of the functional status of hemodialysis patients. J. Bodyw. Mov. Ther..

[33] IBM Corp (2018). IBM SPSS Statistics for Windows; Version 27.0.

[34] Cohen J. (1992). A power primer. Psychol. Bull..

[35] Faul F., Erdfelder E., Lang A.G., Buchner A. (2007). G*Power 3: A flexible statistical power analysis program for the social, behavioral, and biomedical sciences. Behav. Res. Methods.

[36] Benjamin O., Lappin S.L. (2021). End-Stage Renal Disease. StatPearls.

[37] Cabrera V.J., Hansson J., Kliger A.S., Finkelstein F.O. (2017). Symptom management of the patient with CKD: The role of dialysis. Clin. J. Am. Soc. Nephrol..

[38] Kim J.Y., Kim B., Park K.S., Choi J.Y., Seo J.J., Park S.H., Kim C.D., Kim Y.L. (2013). Health-related quality of life with KDQOL-36 and its association with self-efficacy and treatment satisfaction in korean dialysis patients. Qual. Life Res..

[39] Zazzeroni L., Pasquinelli G., Nanni E., Cremonini V., Rubbi I. (2017). Comparison of Quality of Life in Patients Undergoing hemodialysis and peritoneal dialysis: A systematic review and meta-analysis. Kidney Blood Press. Res..

[40] Garcia R.S.A., Lucinda L.M.F., Ramos F.A., Bueno G.S., de Oliveira G.M.R., Bonisson L.S., Silva M.A., Zolli T.I., Pinheiro B.V., Paula R.B. (2017). Factors associated with functional capacity in hemodialysis patients. Artif. Organs.

[41] Johansen K.L., Shubert T., Doyle J., Soher B., Sakkas G.K., Kent-Braun J.A. (2003). Muscle atrophy in patients receiving hemodialysis: Effects on muscle strength, muscle quality, and physical function. Kidney Int..

[42] Yang J., Wahner-Roedler D.L., Zhou X., Johnson L.A., Do A., Pachman D.R., Chon T.Y., Salinas M., Millstine D., Bauer B.A. (2021). Acupuncture for palliative cancer pain management: Systematic review. BMJ Support. Palliat. Care.

[43] Wang Y., Hou Y.Q., Yang J.W., Wang L.Q., Shao J.K., Zou X., Yang N.N., Huang J., Liu C.Z. (2020). Acupuncture of different treatment frequency in postprandial distress syndrome: A pilot randomized clinical trial. Neurogastroenterol. Motil..

[44] Lin L.L., Tu J.F., Wang L.Q., Yang J.W., Shi G.X., Li J.L., Zhang N., Shao J.K., Zou X., Liu C.Z. (2020). Acupuncture of different treatment frequencies in knee osteoarthritis: A pilot randomised controlled trial. Pain.

